# Genetic heterogeneity of residual variance - estimation of variance components using double hierarchical generalized linear models

**DOI:** 10.1186/1297-9686-42-8

**Published:** 2010-03-19

**Authors:** Lars Rönnegård, Majbritt Felleki, Freddy Fikse, Herman A Mulder, Erling Strandberg

**Affiliations:** 1 Statistics Unit, Dalarna University, SE-781 70 Borlänge, Sweden; 2 Department of Animal Breeding and Genetics, Swedish University of Agricultural Sciences, SE-750 07 Uppsala, Sweden; 3 Animal Breeding and Genomics Centre, Wageningen UR Livestock Research, PO Box 65, 8200 AB Lelystad, The Netherlands

## Abstract

**Background:**

The sensitivity to microenvironmental changes varies among animals and may be under genetic control. It is essential to take this element into account when aiming at breeding robust farm animals. Here, linear mixed models with genetic effects in the residual variance part of the model can be used. Such models have previously been fitted using EM and MCMC algorithms.

**Results:**

We propose the use of double hierarchical generalized linear models (DHGLM), where the squared residuals are assumed to be gamma distributed and the residual variance is fitted using a generalized linear model. The algorithm iterates between two sets of mixed model equations, one on the level of observations and one on the level of variances. The method was validated using simulations and also by re-analyzing a data set on pig litter size that was previously analyzed using a Bayesian approach. The pig litter size data contained 10,060 records from 4,149 sows. The DHGLM was implemented using the ASReml software and the algorithm converged within three minutes on a Linux server. The estimates were similar to those previously obtained using Bayesian methodology, especially the variance components in the residual variance part of the model.

**Conclusions:**

We have shown that variance components in the residual variance part of a linear mixed model can be estimated using a DHGLM approach. The method enables analyses of animal models with large numbers of observations. An important future development of the DHGLM methodology is to include the genetic correlation between the random effects in the mean and residual variance parts of the model as a parameter of the DHGLM.

## Background

In linear mixed models it is often assumed that the residual variance is the same for all observations. However, differences in the residual variance between individuals are quite common and it is important to include the effect of heteroskedastic residuals in models for traditional breeding value evaluation [1]. Such models, having explanatory variables accounting for heteroskedastic residuals, are routinely used by breeding organizations today. The explanatory variables are typically non-genetic [2], but genetic heterogeneity can be present and it is included as random effects in the residual variance part of the model.

Modern animal breeding requires animals that are robust to environmental changes. Therefore, we need methods to estimate both variance components and breeding values in the residual variance part of the model to be able to select for animals having smaller environmental variances. Moreover, if genetic heterogeneity is present then traditional methods for predicting selection response may not be sufficient [3,4].

Methods have previously been developed to estimate the degree of genetic heterogeneity. San Cristobal-Gaudy et al. [5] have developed an EM-algorithm. Sorensen & Waagepetersen [6] have applied a Markov chain Monte Carlo (MCMC) algorithm to estimate the parameters in a similar model, which has the advantage of producing model-checking tools based on posterior predictive distributions and model-selection criteria based on Bayes factor and deviances. At the same time, Bayesian methods to fit models with residual heteroskedasticity for multiple breed evaluations [7] and generalized linear mixed models allowing for a heterogenetic dispersion term [8] have been developed. Wolc et al. [9] have studied a sire model, with random genetic effects included in the residual variance, by fitting squared residuals with a gamma generalized linear mixed model.

However, Lee & Nelder [10] have recently developed the framework of double hierarchical generalized linear models (DHGLM). The parameters are estimated by iterating between a hierarchy of generalized linear models (GLM), where each GLM is estimated by iterative weighted least squares. DHGLM give model checking tools based on GLM theory and model-selection criteria are calculated from the hierarchical likelihood (h-likelihood) [11]. Inference in DHGLM is based on the h-likelihood theory and is a direct extension of the hierarchical GLM (HGLM) algorithm [11]. Both the theory and the fitting algorithm are explained in detail in Lee, Nelder & Pawitan [12]. HGLMs have previously been applied in genetics (e.g. [13,14]) but animal breeding models have not been studied using DHGLM.

A user-friendly version of DHGLM has been implemented in the statistical software package GenStat [15]. To our knowledge, DHGLM has only been applied on data with relatively few levels in the random effects (less than 100), whereas models in animal breeding applications usually have a large (*>>*100) number of levels in the random effects. The situation is most severe for animal models, where the number of levels in the random genetic effect can be greater than the number of observations, and the number of observations often exceeds 10^6^. Thus, a method to estimate genetic heterogeneity of the residual variance in animal models with a large number of observations is desirable.

The aim of the paper is to study the potential use of DHGLM to estimate variance components in animal breeding applications. We evaluate the DHGLM methodology by means of simulations and compare the DHGLM estimates with MCMC estimates using field data previously analyzed by Sorensen & Waagepetersen [6].

## Materials and methods

In this section we start by defining the studied model. Thereafter, we review the development of GLM-based algorithms to fit models with predictors in the residual variance. The DHGLM algorithm is presented and we continue by showing how a slightly modified version of DHGLM can be implemented in ASReml [16]. Thereafter, we describe our simulations and the data from Sorensen & Waagepetersen [6] that we reanalyze using DHGLM.

We consider a model consisting of a mean part and a dispersion part. There is a random effect *u *in the mean part of the model and a random effect *u*_*d *_in the dispersion part (subscript *d *is used to denote a vector or a matrix in the dispersion part of the model). The studied trait *y *conditional on *u *and *u*_*d *_is assumed to be normal. The mean part of the model is(1)

with a linear predictor(2)

The dispersion part of the model is specified as(3)

with a linear predictor(4)

Let *n *be the number of observations (i.e. the length of *y*), and let *q *be the length of *u *and *q*_*d *_the length of *u*_*d*_. Normal distributions are assumed for *u *and *u*_*d*_, i.e. *u *~*N *(0, **I**_*q *_) and *u*_*d *_~*N *(0, ), where **I**_*q *_and  are identity matrices of size *q *and *q*_*d*_, respectively. The fixed effects in the mean and dispersion parts are *b *and *b*_*d*_, respectively. In the present paper, *u *and *u*_*d *_are treated as non-correlated so that(5)

We allow for more than one random effect in the mean and dispersion parts of the model. Furthermore, it is possible to have a random effect with a given correlation structure. The correlation structure of *u *can be included implicitly by modifying the incidence matrix **Z **[12]. If we have an animal model, for instance, the relationship matrix **A **can be included by multiplying the incidence matrix **Z **with the Cholesky factorization of **A**. Cholesky factorization of **A **may, however, lead to reduced sparsity in the mixed model equations.

Distributions other than normal for the outcome *y *can be modelled in the HGLM framework, as well as non-normal distributions for the random effects, but these will not be considered here. HGLM theory in a more general setting is given in the Appendix.

### Linear models with fixed effects in the dispersion

We start by considering a linear model with only fixed effects both in the mean and dispersion parts. Using GLM to fit these models has been applied for several decades [17]. Maximum likelihood estimates for the fixed effects in the dispersion part can be achieved by using a gamma GLM with squared residuals as response.

The basic idea is that if the fixed effects *b *in the mean part of the model were given (known without uncertainty) then the squared residuals are  (for observation *i*), i.e. gamma distributed with a scale parameter equal to 2 (with  = *ϕ*_*i *_and ). The squared residuals may be fitted using a GLM [18] having a gamma distribution together with a log link function. Hence, a linear model is fitted for the mean part of the model, such that(6)

where *ϕ*_*i *_are estimated from the gamma GLM with(7)(8)

However, *b *is estimated and we only have the predicted residuals . The expectation of  is not equal to *ϕ*_*i *_and a REML adjustment is required to obtain unbiased estimates. This is achieved by using the leverages *h*_*i *_from the mean part of the model. The fitting algorithm gives REML estimates [19] if we replace eq. 7 by(9)

and use weights (1 - *h*_*i*_)/2, (since [12]). The leverage *h*_*i *_for observation *i *is defined as the *i*:th diagonal element of the hat matrix [20](10)

Here, *W *is the weight matrix for the linear model in eq. 6, i.e. *w*_*i *_= 1/. The estimation algorithm iterates between the fitting procedures of eq. 9 and eq. 6, and the diagonal elements *w*_*i *_in *W *are updated on each iteration using , the predicted values from the dispersion model. Note that this algorithm gives exact REML estimates and is not an approximation [19,21,22].

### Linear mixed models and HGLM

Here, a linear mixed model with homoskedastic residuals is considered. Lee & Nelder [11] have shown that REML estimates for linear mixed models can be obtained by using a hierarchy of GLM and augmented linear predictors. An important part of the fitting procedure is to present Henderson's [23] mixed model equations in terms of a weighted least squares problem. This is achieved by augmenting the response variable *y *with the expectation of *u*, where *E*(*u*) = **0**.

The linear mixed model

may be written as an augmented weighted linear model(11)

where

The variance-covariance matrix of the augmented residual vector is given by

The estimates from weighted least squares are given by

This is identical to Henderson's mixed model equations where the left hand side can be verified to be(12)

The variance component  is estimated by applying a gamma GLM to the response /(1 - *h*_*i*_) with weights (1 - *h*_*i*_)/2, where the index *i *goes from 1 to *n*. Similarly,  is estimated by applying a gamma GLM to the response /(1 - *h*_*j*_) with weights (1 - *h*_*j*_)/2, where the index *j *goes from 1 to *q *and *h*_*j *_comes from the last *q *leverages of the augmented model. The augmented model gives leverages equal to the diagonal elements of(13)

Leverages with values close to 1.0 indicate severe imbalance in the data. For the last *q *diagonal elements in **H**, 1-*h*_*j *_is equivalent to the reliabilities [24] of the BLUP values of *u*.

This algorithm gives exact REML estimates for a linear mixed model with normal *y *and *u *[12].

### Linear mixed models with fixed effects in the dispersion within the HGLM framework

Since the linear mixed model can now be reformulated as a weighted least squares problem, we can use the fitting algorithm for weighted least squares described above to estimate *b*, *u *together with the fixed effects in the dispersion part of the model *b*_*d*_, as well as the variance component in the mean part of the model .

This HGLM estimation method has previously been used in genetics to analyse lactation curves with heterogeneous residual variances over time [14], where it was shown that the algorithm gives REML estimates. A recently developed R [25] package **hglm **[26] is also available on CRAN http://cran.r-project.org, which enables fitting of fixed effects in the residual variance.

### Double HGLM

Now we extend the model further and include random effects in the dispersion part. A gamma GLM is fitted using the linear predictor(14)

By applying the augmented model approach similar to eq. 11 also to the dispersion part of the model we obtain a double HGLM (DHGLM)(15)

where(16)(17)

Here,  denotes a vector of ones so that its logarithm matches the expectation of *u*_*d*_, where *E*(*u*_*d*_) = 0 (see Table 7.1 in [12]).

The mean part of the model is fitted as described in the previous section. The dispersion part of the model is fitted by using an augmented response vector *y*_*d *_based on the squared residuals from eq. 11

with weights

The vector of individual deviance components *d*_*d *_is subsequently used to estimate  by fitting a gamma GLM to the response *d*_*d*, *j*_/(1 - *h*_*d*, *j*_) with weights (1 - *h*_*d*, *j*_)/2, where *d*_*d*, *j *_is the *j*:th component of *d*_*d *_and *h*_*d*, *j *_is the *j*:th element of the last *q*_*d *_leverages.

#### Algorithm overview

The fitting algorithm is implemented as follows.

1. Initialize ,  and *W*.

2. Estimate *b *and *u *by fitting the model for the mean using eq. 11 (i.e. Henderson's mixed model equations) and calculate the leverages *h*_*i*_.

3. Estimate  by fitting a gamma GLM to the response /(1 - *h*_*j*_) with weights (1 - *h*_*j*_)/2, where *h*_*j *_are the last *q *diagonal elements of the hat matrix **H**.

4. Estimate *b*_*d *_and *u*_*d *_from eq. 15 (using Henderson's mixed model equations) with , and calculate the deviance components *d*_*d *_and leverages *h*_*d*_

5. Estimate  by fitting a gamma GLM to the response *d*_*d*, *j*_/(1 - *h*_*d*, *j*_) with weights (1 - *h*_*d*, *j*_)/2

6. Update the weight matrix *W *as(18)

7. Iterate steps 2-6 until convergence

We have described the algorithm for one random effect in the mean and dispersion parts of the model but extending the algorithm for several random effects is rather straightforward [12]. The algorithm has been implemented in GenStat [12,15] where the size of the mixed model equations is limited and thus could not be used in our analysis. Hence, we implemented the algorithm using PROC REG in SAS^®^, but found that it was too time consuming to be useful on large data sets. A faster version of the algorithm was therefore implemented using the ASReml software [16]. As described below, the ASReml implementation uses penalized quasi-likelihood (PQL) estimation in a gamma GLMM.

### DHGLM implementation using penalized quasi likelihood estimation

PQL estimates, for a generalized linear mixed model (GLMM), are obtained by combining iterative weighted least squares and a REML algorithm applied on the *adjusted dependent variable *(which is calculated by linearizing the GLM link function) [27]. For instance, the GLIMMIX procedure in SAS^® ^iterates between several runs of PROC MIXED and thereby produces PQL estimates.

By iterating between a linear mixed model for the mean and a gamma GLMM for the dispersion part of the model using PQL, a similar algorithm as the one described above can be implemented. If the squared residuals of the adjusted dependent variable were used in the DHGLM (as described in the previous section) to calculate  instead of the deviance components, the algorithm would produce PQL estimates [12]. Both of these two alternatives to estimate  in a gamma GLMM give good approximations [12,27]. Hence, both methods are expected to give good approximations of the parameter estimates in a DHGLM, but, to our knowledge, the exact quality of these approximations has not been investigated, so far.

ASReml uses PQL to fit GLMM and has the nice property of using sparse matrix techniques to calculate the leverages *h*_*i*_. Although we used ASReml to implement a PQL version of the DHGLM algorithm, any REML software that uses sparse matrix techniques and produces leverages should be suitable.

Let *h*_*asreml *_be the *hat values *calculated in ASReml and stored in the .yht output file. They are defined in the *ASReml User Guide *[16] as the diagonal elements of [**X**, **Z**](**T**^*t*^*W***T**)^-1 ^[**X**, **Z**]^*t*^. So, the leverages *h *are equal to *W*_*asreml*_*·h*_*asreml *_where *W*_*asreml *_is the diagonal matrix of prior weights specified in ASReml and  is the estimated residual variance.

The PQL version of the DHGLM algorithm was implemented as follows.

1. Initialize *W *= **I**_*n*_

2. Estimate *b*, *u *and  by fitting a linear mixed model to the data *y *and weights *W*

3. Calculate *y*_*d*, *i *_= /(1 - *h_i_*) and 

4. Estimate *b*_*d*_, *u*_*d *_and  by fitting a weighted gamma GLM with response *y*_*d *_and weights *W*_*d*_.

5. Update *W *= *diag*()^-1^, where  are the predicted values from the model in Step 4.

6. Iterate steps 2-5 until convergence.

Convergence was assumed when the change in variance components between iterations was less than 10^-5^. The algorithm is quite similar to the one used by Wolc et al. [9] to fit a sire model with genetic heterogeneity in the residual variance, except that they did not make the leverage corrections to the squared residuals. Including the leverages in the fitting procedure is important to obtain acceptable variance component estimates in animal models and also for imbalanced data.

### Simulation study

To test whether the DHGLM approach gives unbiased estimates for the variance components, we simulated 10,000 observations and a random group effect. The number of groups was either 10, 100 or 1000. An observation for individual *i *with covariate *x*_*k *_belonging to group *l *was simulated as: *y*_*ikl *_= 1.0 + 0.5*x*_*k *_+ *u*_*l *_+ *e*_*ikl*_, where the random group effects are iid with *u*_*l *_~*N *(0, ), and the residual effect was sampled from *N*(0, *V *(*e*_*ikl*_)) with: *V *(*e*_*ikl*_) = *exp*(0.5 + 1.5*x*_*d*, *k *_+ *u*_*d*, *l*_), where *x*_*d*, *k *_is a covariate. The covariates *x*_*k *_and *x*_*d*, *k *_were simulated binary to resemble sex effects. Furthermore, *u*_*d*, *l*_~*N *(0, ) with *cov*(*u*_*l*_, *u*_*d*, *l*_) = *ρσ*_*u*_*σ*_*d*_. The simulated variance components were  = 0.5 and  = 1.0, whereas the correlation *ρ *was either 0 or -0.5. The value of  = 1.0 gives a substantial variation in the simulated elements of *u*_*d*_, where a one standard deviation difference between two values *u*_*d*, *l *_and *u*_*d*, *m *_increases the residual variance 2.72 times. The simulated value of  was chosen to be quite large, compared to the residual variance, because large values of  should reveal potential bias in DHGLM estimation using PQL [27]. The average value of the residual variance was 3.5. We replicated the simulation 20 times and obtained estimates of variance components using the PQL version of DHGLM.

### Re-analyses of pig litter size: data and models

Pig litter size has been previously analyzed by Sorensen & Waagepetersen [6] using Bayesian methods, and the data is described therein. The data includes 10,060 records from 4,149 sows in 82 herds. Hence, repeated measurements on sows have been carried out and a permanent environmental effect of each sow has been included in the model. The maximum number of parities is nine. The data includes the following class variables: herd (82 classes), season (4 classes), type of insemination (2 classes), and parity (9 classes). The data is highly imbalanced with two herds having one observation and 13 herds with five observations or less. The ninth parity includes nine observations.

Several models has been analyzed by Sorensen & Waagepetersen [6] with an increasing level of complexity in the model for the residual variance and with the model for the mean *y *= **Xb **+ **Wp **+ **Za **+ **e **varying only through the covariance matrix *V *(**e**). Here *y *is litter size (vector of length 10,060), **b **is a vector including the fixed effects of herd, season, type of insemination and parity, and **X **is the corresponding design matrix (10,060 × 94), **p **is the random permanent environmental effect (vector of length 4,149), **W **is the corresponding incidence matrix (10,060 × 4,149) and *V *(**p**) = **I **, **a **is the additive genetic random effect, **Z **is the corresponding incidence matrix (10,060 × 6,437) and *V *(**a**) = **A ** where **A **is the additive relationship matrix. Hence the LHS of the mixed model equations is of size 10,680 × 10,680.

The residual variance **e **was modelled as follows.

#### Model I: Homogeneous variance

where *b*_0 _is a common parameter for all *i*.

#### Model II: Fixed effects in the linear predictor for the residual variance

In this model each parity and insemination type has its own value for the residual variance

where **b**_*d *_is a parameter vector including effects of parity and type of insemination, and **x**_*d*, *i *_is the *i*:th row in the design matrix **X**_*d*_.

#### Model III: Random animal effects together with fixed effects in the linear predictor for the residual variance

where **z**_*i *_is the *i*:th row of **Z **and **a**_*d *_is a random animal effect with **a**_*d *_~.

#### Model IV: Both permanent environmental effects and animal effects in the linear predictor for the residual variance

where **w**_*i *_is the *i*:th row of **W **and **p**_*d *_is a random permanent environmental effect with **p**_*d *_~.

These four models are the same as in [6] with the difference that we do not include a correlation parameter between **a **and **a**_*d *_in our analysis.

## Results

### Simulations

The DHGLM estimation produced acceptable estimates for all simulated scenarios (Table 1), with standard errors being large for scenarios with few groups, i.e. for a small number of elements in *u *and *u*_*d*_. In animal breeding applications, the length of *u *and *u*_*d *_is usually large and we can expect the variance components to be accurately estimated. The estimates were not impaired by simulating a negative correlation between *u *and *u*_*d *_although a zero correlation was assumed in our fitting algorithm.

**Table 1:** Estimated variance components in the model of the mean and the residual variance using DHGLM. The variance of the random effects in the mean and residual parts of the model are  and , respectively; results given as mean (s.e.) of 20 replicates

		Simulated values			Estimates	
No. groups	Obs. per group			*ρ*		
1000	10	0.5	1.0	0.0	0.50	1.06
					(0.03)	(0.06)
1000	10	0.5	1.0	-0.5	0.47	1.07
					(0.03)	(0.05)
100	100	0.5	1.0	0.0	0.51	0.98
					(0.01)	(0.03)
100	100	0.5	1.0	-0.5	0.49	1.01
					(0.01)	(0.04)
10	1000	0.5	1.0	0.0	0.53	0.80
					(0.04)	(0.10)
10	1000	0.5	1.0	-0.5	0.42	1.03
					(0.04)	(0.10)

### Analysis of pig litter size data

The DHGLM estimates and Bayesian estimates (i.e. posterior mean estimates from [6]) were identical for the linear mixed model with homogeneous variance (Model I) and were very similar for Model II where fixed effects are included in the residual variance part of the model (Table 2). For Model III and IV, including random effects in the residual variance part of the model, the DHGLM estimates deviated from the Bayesian point estimates for the mean part of the model. Nevertheless, the DHGLM estimates were all within the 95% posterior intervals obtained by Sorensen & Waagepetersen [6]. The differences were likely due to the fact that the genetic correlation *ρ *was not included as a parameter in the DHGLM approach. The correspondence between the two methods for the variance components in the residual variance was very high.

**Table 2:** Comparison between DHGLM estimates and the estimates obtained by Sorensen & Waagepetersen [6] (referred to as S&W 2003 below)

				Model for residual variance					
		Mean model		Fixed effects			Variances		
Model				*b*_0_	*δ*_*ins*_	*δ*_*par*_			*ρ*
I	DHGLM	1.40	0.60	2.00					
	S&W 2003	1.40	0.60	2.00					
II	DHGLM	1.38	0.73	1.87	-0.15	0.34			
	S&W 2003	1.37	0.71	1.87	-0.15	0.34			
III	DHGLM	1.35	0.53	1.73	-0.17	0.32	0.13		*
	S&W 2003	1.58	0.60	1.78	-0.16	0.34	0.11		-0.57
IV	DHGLM	1.36	0.44	1.72	-0.17	0.32	0.09	0.06	*
	S&W 2003	1.62	0.60	1.77	-0.17	0.35	0.09	0.06	-0.62

*b*_0 _is the intercept term in the model for the residual variance

*δ*_*ins *_is the fixed effect of insemination in the model for the residual variance

*δ*_*par *_is the fixed effect for the difference in first and second parity in the model for the residual variance

*The correlation between *a *and *a*_*d *_was not estimated with DHGLM

**Figure 1:**
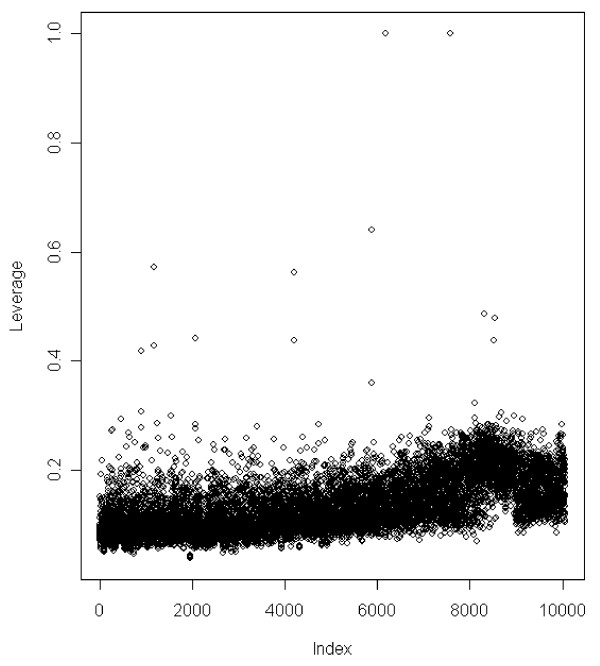
**Leverages for the mean part of the model**. Leverages *h*_*i *_for the 10,060 observations of pig litter size for Model IV with both permanent environmental and animal random effects included in the residual variance part of the model.

The data was unbalanced with few observations within some herds, i.e. two herds contain only single observations. The observations from these two herds have leverages equal to 1.0 (Figure 1) and do not add any information to the model. Leverage plots can be a useful tool in understanding results from models in animal breeding and our results show that they illustrate important aspects of imbalance.

For Model IV, the DHGLM algorithm implemented using ASReml converged in 10 iterations and the computation time was less than 3 minutes on a Linux server (with eight 2.66 GHz quad core CPUs and 16 Gb memory).

## Discussion

We have shown that DHGLM is a feasible estimation algorithm for animal models with heteroskedastic residuals including both genetic and non-genetic heterogeneity. Furthermore, a fast version of the algorithm was implemented using the ASReml [16] software. Hereby, estimation of variance components in animal models with a large number of observations is possible. We have explored the accuracy and speed of variance component estimation using DHGLM but the algorithm also produces estimated breeding values. It is important to consider heteroskedasticity in traditional breeding value evaluation, because failing to do so leads to suboptimal selection decisions [2,7,28], and models with genetic heterogeneity is important when aiming at selecting robust animals [3]. Variance component estimation and breeding value evaluation in applied animal breeding are typically based on large data sets, and we therefore expect that the proposed DHGLM algorithm could be of wide-spread use in future animal breeding programs. Especially, since breeding organizations usually have a stronger preference for traditional REML estimation than in the previously proposed Bayesian methods [6-8].

We have focused on traits that are normal distributed (conditional on the random effects). The HGLM approach permits modelling of traits following any distribution from the exponential family of distributions, e.g. normal, gamma, binary or Poisson. Equation 11 is then re-formulated by specifying the distribution and by using a link function *g*(.) so that *g*(*μ*) = **T***δ *(see Appendix). In this more general setting, the individual deviance components [18] are used instead of the squared residuals to estimate the variance components. HGLM gives only approximate variance component estimates if the response is not normal distributed. For continuous distributions, including gamma, the approximation is very good. For discrete distributions, such as binomial and Poisson, the approximation can be quite poor, but higher-order corrections based on the h-likelihood are available [13]. Kizilkaya & Tempelman [8] have developed Bayesian methods to fit generalized linear mixed models with heteroskedastic residuals and genetic heterogeneity. This method is more flexible, since a wider range of distributions for the residuals can be modeled, but it is much more computationally demanding.

An important feature of the DHGLM algorithm is that it requires calculation of leverages. Wolc et al. [9] have fitted a generalized linear mixed model to the squared residuals of a sire model without adjusting for the leverages. However, for models with animal effects it is essential to include the leverage adjustments. The effects of adjusting for the leverages, or not, are similar to the effects of using REML instead of ML to fit mixed linear models, where ML gives biased variance component estimates and the estimates are more sensitive to data imbalance [12]. Moreover, the leverages can be a useful tool to identify important aspects of data imbalance (as shown in Figure 1).

DHGLM estimation is available in the user-friendly environment of GenStat [12,15]. Fitting DHGLM in GenStat is possible for models with up to 5,000 equations in the mixed model equations (results not shown). Hence, the GenStat version of DHGLM is suitable for sire models but not for animal models if the number of observations is large. An advantage of GenStat, however, is that it produces model-selection criteria for DHGLM based on the h-likelihood. Nevertheless, it does not include estimation of the correlation parameter *ρ*.

Simple methods based on linear mixed models have been proposed [9,29] to estimate *ρ*, but an unbiased and robust estimator for animal models still requires further research. To our knowledge, methods to estimate *ρ *within the DHGLM framework has not been developed yet. An important future development of the DHGLM is, therefore, to incorporate *ρ *in the model and to study how other parameter estimates are affected by the inclusion of *ρ*. Another essential development of such a model would be to derive model-selection criteria based on the h-likelihood (see [12]).

## Competing interests

The authors declare that they have no competing interests.

## Authors' contributions

ES initiated the study. LR was responsible for the analyses and writing of the paper. MF implemented a first version of the DHGLM algorithm in R and performed part of the analyses. FF and HM initiated the idea of implementing DHGLM using ASReml. All authors were involved in reading and writing the paper.

## Appendix

### H-likelihood theory

Here we summarize the h-likelihood theory for HGLM according to the original paper by Lee & Nelder [11], which justifies the estimation procedure and inference for HGLM. H-likelihood theory is based on the principle that HGLMs consist of three objects: data, fixed unknown constants (parameters) and unobserved random variables (unobservables). This is contrary to traditional Bayesian models which only consist of data and unobservables, while a pure frequentist's model only consists of the data and parameters.

The h-likelihood principle is not generally accepted by all statisticians. The main criticism for the h-likelihood has been non-invariance of inference with respect to transformation. This criticism would be appropriate if the h-likelihood was merely a joint likelihood of fixed and random effects. However, the restriction that the random effects occur linearly in the linear predictor of an HGLM is implied in the h-likelihood, which guarantees invariance [30].

Let *y *be the response and *u *an unobserved random effect. A hierarchical model is assumed so that *y*|*u *~*f*_*m*_(*μ*, *ϕ*) and *u *~*f*_*d*_(*ψ*, *λ*) where *f*_*m *_and *f*_*d *_are specified distributions for the mean and dispersion parts of the model. Furthermore, it is assumed that the conditional (log-)likelihood for *y *given *u *has the form of a GLM likelihood(19)

where *θ' *is the canonical parameter, *ϕ *is the dispersion term, *μ' *is the conditional mean of *y *given *u *where *η' *= *g*(*μ'*), i.e. *g*(.) is a link function for the GLM. The linear predictor for *μ' *is given by *η' *= *η *+ *v *where *η *= *Xb*. The dispersion term *ϕ *is connected to a linear predictor *X*_*d*_*b*_*d *_given a link function *g*_*d*_(.) with *g*_*d*_(*ϕ*) = *X*_*d*_*b*_*d*_.

It is not feasible to use a classical likelihood approach by integrating out the random effects for this model (except for a few special cases including the case when *f*_*m *_and *f*_*d *_are both normal). Therefore a h-likelihood is used and is defined as(20)

where *l*(*α*; *v*) is the log density for *v *with parameter *α *and *v *= *v*(*u*) for some strict monotonic function of *u*.

The estimates of *b *and *v *are given by  = 0 and  = 0. The dispersion components are estimated by maximizing the adjusted profile h-likelihood(21)

where *H *is the Hessian matrix of the h-likelihood.

Lee & Nelder [11] showed that the estimates can be obtained by iterating between a hierarchy of GLM, which gives the HGLM algorithm. The h-likelihood itself is not an approximation but the adjusted profile h-likelihood given above is a first-order Laplace approximation to the marginal likelihood and gives excellent estimates for non-discrete distributions of *y*. For binomial and Poisson distributions higher-order approximations may be required to avoid severely biased estimates [12].

### Double Hierarchical Generalized Linear Models

Here we present the h-likelihood theory for DHGLM and refer to the paper on DHGLM by Lee & Nelder [10] for further details.

For DHGLM it is assumed that conditional on the random effects *u *and *u*_*d*_, the response *y *satisfies *E*(*y*|*u*, *u*_*d*_) = *μ *and *var*(*y*|*u*, *u*_*d*_) = *ϕV*(*μ*), where *V*(*μ*) is the GLM variance function, i.e. *V*(*μ*) ≡ *μ*^*k *^where the value of *k *is completely specified by the distribution assumed for *y*|*u*, *u*_*d *_[18]. Given *u *the linear predictor for *μ *is *g*(*μ*)= **X***b *+ **Z***v*, and given *u*_*d *_the linear predictor for *ϕ *is *g*_*d*_(*ϕ*) = **X**_*d*_*b*_*d *_+ **Z**_*d*_*v*_*d*_. The h-likelihood for a DHGLM is(22)

where *l*(*α*_*d*_; *v*_*d*_) is the log density for *v*_*d *_with parameter *α*_*d *_and *v*_*d *_= *v*_*d*_(*u*_*d*_) for some strict monotonic function of *u*_*d*_.

In our current implementation we use an identity link function for *g*(.) and a log link for *g*_*d*_(.).

Furthermore, we have *v *= *u *and *v*_*d *_= *u*_*d *_such that *μ *= **X***b *+ **Z***u *and *log*(*ϕ*) = **X**_*d*_*b*_*d *_+ **Z**_*d*_*u*_*d*_. We restricted our analysis to a normally distributed trait for *var*(*y*|*u*, *u*_*d*_) such that *var*(*y*|*u*, *u*_*d*_) = *ϕ*, and we also assumed *u *and *u*_*d *_to be normal.

The performance of DHGLM in multivariate volatility models (i.e. multiple time series with random effects in the residual variance) has been studied in an extensive simulation study [31]. The maximum likelihood estimates (MLE) for this multivariate normal-inverse-Gaussian model were available and the authors could therefore compare the MLE with the DHGLM estimates. The estimates were close to the MLE for all simulated cases and the approximation improved as the number of time series increased from one to eight. Hence, for the studied time-series model, the DHGLM estimates improve as the number of observations increases, given a fixed number of elements in *u*_*d*_. These results highlight that DHGLM is an approximation, but that the approximation can be expected to be satisfactory when *y*|*u*, *u*_*d *_is normally distributed.
